# Both Talin-1 and Talin-2 correlate with malignancy potential of the human hepatocellular carcinoma MHCC-97 L cell

**DOI:** 10.1186/s12885-016-2076-9

**Published:** 2016-01-28

**Authors:** Kun-peng Fang, Wei Dai, Yan-Hong Ren, Ye-Chuan Xu, She-min Zhang, Ye-Ben Qian

**Affiliations:** The People’s Hospital, Xuancheng City, Auhui Province China; First Affiliated Hospital of Anhui Medical University, Hefei, 230032 Anhui Province China

**Keywords:** Hepatocellular carcinoma (HCC), Talin-1, Talin-2, Migration, Invasion, Cell cycle arrest

## Abstract

**Background:**

Talin-1 (TLN-1) and TLN-2 are implicated in many cellular processes, but their roles in hepatocellular carcinoma (HCC) remain unclear. This study aimed to assess cell cycle distribution, anoikis, invasion and migration in human HCC MHCC-97 L cells.

**Methods:**

MHCC-97 L cells, which highly express TLN-1, were transduced with TLN-1 shRNA (experimental group) or scramble shRNA (negative control group); non-transduced MHCC-97 L cells were used as blank controls. TLN-1 and TLN-2 mRNA and protein levels were detected by real-time RT-PCR and western blot, respectively. Then, cell cycle distribution and anoikis were assessed by flow cytometry. In addition, migration and invasion abilities were assessed using Transwell and cell scratch assays. Finally, a xenograft nude mouse model was established to further assess cell tumorigenicity.

**Results:**

Compared with the blank and negative control groups, TLN-1/2 mRNA and protein levels were significantly reduced in the experiment group. TLN-1/2 knockdown cells showed significantly more cells in the G0/G1 phase (79.24 %) in comparison with both blank (65.36 %) and negative (62.69 %) control groups; conversely, less cells were found in G2/M and S phases in the experimental group compared with controls. Moreover, anoikis was enhanced (*P* < 0.05), while invasion and migration abilities were reduced (*P* < 0.05) in TLN-1/2 knockdown cells compared with controls. TLN-1/2 knockdown inhibited MHCC-97 L cell migration (Percentage of wound healing area: experimental group: 32.6 ± 0.7 % *vs*. negative controls: 50.1 ± 0.6 % and blank controls: 53.6 ± 0.6 %, both *P* < 0.01). Finally, the tumors obtained with TLN-1/2 knockdown cells were smaller (*P* < 0.05) compared with controls.

**Conclusion:**

Both TLN-1 and TLN-2 levels correlate with tumorigenicity in human HCC, indicating that these molecules constitute important molecular targets for the diagnosis and/or treatment of HCC.

## Background

Hepatocellular carcinoma (HCC) is a major health problem [1]. Recent studies have reported an incidence of 780,000cases/year for HCC, with most individuals diagnosed at intermediate or advanced disease stages, where curative approaches are often not feasible [2]. Although there is no definitively curative treatment for HCC, multiple therapeutic and management options exist with various advantages and disadvantages [3]: the surgical options include resection, transplantation and ablation; in addition, radiation therapy and biologic agents such as Sorafenib have been proposed.

Two talin genes present in vertebrates, namely Talin-1 (TLN-1) and Talin-2 (TLN-2), encode proteins with 74 % sequence identity; TLN-2 is the ancestral gene, with TLN-1 arising by gene duplication early in the chordate lineage [4–7]. Despite the high homology between both talin proteins, differences in the remaining amino acids may affect protein function [8, 9]. TLN-1 is primarily expressed in the kidney, liver, spleen, stomach, lung, and vascular smooth muscle [5, 10–13]. Additionally, TLN-2 mRNA expression is most abundant in the heart, brain, and skeletal muscle [5, 13]. However, other recent western blotting data and expression studies with a mouse gene trap line have suggested that TLN-2 may be more widely expressed [5, 14, 15].

TLN-1 is a cytoskeletal protein of 270 kDa that plays a pivotal role in regulating the activity of the integrin family of cell adhesion proteins by coupling them to F-actin [16, 17]. TLN-1 is also a focal adhesion protein that binds to multiple adhesion molecules, including integrins, vinculin, focal adhesion kinase (FAK), and actin. Moreover, TLN-1 plays an essential role in integrin activation [18, 19]. Upon activation, integrins increase the functional interaction between cells and the extracellular matrix (ECM), thus serving as bidirectional transducers of extracellular and intracellular signals, ultimately regulating adhesion, proliferation, anoikis, survival, and tumor progression [18–22]. TLN-1 overexpression has been reported to enhance prostate cancer cell adhesion, migration, and invasion by activating survival signals and conferring resistance to anoikis [19]. TLN-1 overexpression could serve as a diagnostic marker for aggressive phenotypes and a potential target for treating oral squamous cell carcinoma (OSCC) [23]. In addition, kinesin family member 14 (KIF14) and TLN-1 expression levels predict a better outcome after cytotoxic chemotherapy, and inhibition of these genes sensitizes triple-negative breast cancer (TNBC) cells to therapeutic intervention [24]. In a retrospective study of banked tissue samples, alpha-actinin and TLN were found to be completely absent in both endometriosis and endometrioid carcinomas [12, 25]. In HCC research, Chinese investigators have observed that TLN-1 protein and mRNA levels in HCC tissues are significantly lower than those in the adjacent non-cancerous tissues [12]. However, Japanese investigators have reported that TLN-1 is upregulated in HCC [17], and Egyptian studies showed that TLN-1 serum levels in HCC patients are significantly higher [26].

To date, the role and function of TLN-2, as a homologous gene of TLN-1, in tumors remains unclear. Studies have demonstrated that the N-terminal TLN-2 FERM domain binds to β1D-integrin with a higher affinity than that of TLN-1 [27, 28]. Studies using cultured cells have clearly established that TLN-2 can compensate for the loss of TLN-1, supporting cell spreading and focal adhesion (FA) assembly in TLN-1 knockout or knockdown cells [27, 29, 30]. TLN-1 knockout is embryonic lethal at gastrulation in mice, whereas TLN-2 knockout mice are viable and fertile [6, 7, 15].

Previously, we showed that TLN-1 and TLN-2 mRNA and protein expressions differ significantly among five human HCC cell lines and the human normal liver LO_2_ cell line [8]; indeed, TLN-1 and TLN-2 expression levels might be related to invasion and migration in human hepatocellular carcinoma (HCC). To further assess the role of TLN-1 and TLN-2 in HCC, MHCC-97 L cells, which highly express TLN-1, were selected for gene knockdown using RNA interference. The effects of TLN-1/2 silencing on HCC malignancy potential in vivo were also evaluated.

## Methods

The protocols in this study were approved by the Ethics Committee of Anhui Medical University. Animal testing was performed in accordance with the international guiding principles for biomedical research.

### Cell culture

Human hepatocellular carcinoma MHCC-97 L cells were provided by the GanDanYi Experiment Center of Huashan Hospital affiliated to Fudan University, Shanghai (China), and cultured in Dulbecco’s modified Eagle’s medium (DMEM) (Invitrogen, Carlsbad, CA, USA) containing 10 % fetal bovine serum (FBS) (Invitrogen, Carlsbad, CA, USA) and antibiotics (penicillin G/streptomycin, 50 μg/ml) (Sangon Biotech Co., Ltd., Shanghai, China), in a humid environment at 37 °C with 95 % normal air and 5 % CO_2_.

### Lentivirus-mediated stableTLN-1 knockdown in the MHCC-97 L cells

A shRNA sequence targeting the TLN-1 gene (GenBank. No. NM_006289.3) (5’-GCTCGAGATGGCAAGCTTAAA-3’) and one nonspecific sequence 5’-TTCTCCGAACGTGTCACGTTTC-3’ (scramble shRNA) used as the negative control which did not have any homology with the target gene were designed and packaged into Lentivirus and transduced into 293 T cells by Shanghai GenePharma Co., Ltd (China).

For TLN-1 silencing, 0.5 × 10^5^ MHCC-97 L cells were plated in 24-well plates in complete culture medium for 24 h. Then, cells were transduced for 72 h with lentivirus-mediated TLN-1-shRNA and scramble shRNA, respectively (Shanghai GenePharma Co., Ltd, China), under puromycin selection. Afterwards, the transduced cells were cultured in DMEM containing 10 % FBS and 3 μg/ml of puromycin for 12 days (changing the medium every 3 days), and stably transduced cells were obtained. TLN-1 mRNA and protein levels were assessed by real-time RT-PCR and western blot.

### Real-time RT-PCR

Total RNA was extracted using RNeasy Miniprep Kit (Sangon Biotech Co., Ltd., Shanghai, China) according to the manufacturer’s instructions, and treated with Turbo DNase (Ambion, Austin, TX, USA). RNA purity was determined by measuring absorbance at 260 and 280 nm (A260/280). DNase-treated total RNA (1 μg) was reverse-transcribed using Superscript III reverse transcriptase (Invitrogen, Carlsbad, CA, USA) and 500 ng of random primers (Promega, Madison, WI, USA). Glyceraldehyde 3-phosphate dehydrogenase (GAPDH) was used as an internal control. The following primer pairs were used for TLN-1, TLN-2 and GAPDH (http://www.rtprimerdb.org/): TLN-1: sense, 5’- TGTAGAGGAGCACGAGACGC -3’ and anti-sense, 5’- AAGGAGACAGGGTGGGAGC -3’; TLN-2: sense, 5’-CTGAGGCTCTTTTCACAGCA-3’ and anti-sense, 5’-CTCATCTCATCTGCCAAGCA-3’; GAPDH: sense: 5’-CATGAGAAGTATGACAACAGCCT-3’ and anti-sense, 5’- AGTCCTTCCACGATACCAAAGT-3’. Relative gene expression was quantified by real-time PCR using SYBR Premix Ex Taq™ II (TaKaRa Bio, Dalian, China) on a Lightcycler 480 Real-Time PCR System (Roche Diagnostics, Meylan, France). Reactions were carried out with initial denaturation (95 °C for 3 min) followed by 40 cycles of 95 °C for 12 s, 62 °C for 30 s, and 72 °C for 30 s. The cycle threshold (Ct) was defined as the number of cycles required for the fluorescent signal to cross the threshold. First, ΔCt = Ct _Gene_ - Ct _GAPDH_. Then, ΔΔCt = ΔCt _treated_ - ΔCt _control_. Ratios were derived as proposed elsewhere [31].

### Western blotting

Cells were lysed in M-PER Mammalian Protein Extraction Reagent (Thermo, USA), and equal amounts of protein were resolved by 6 % sodium dodecyl sulfate-polyacrylamide gel electrophoresis (SDS-PAGE) and blotted onto polyvinylidene difluoride (PVDF) membranes (Millipore). The following specific primary antibodies were used: anti-TLN-1(ab78291, Abcam, Cambridge, MA, USA), anti-TLN-2 (ab105458, Abcam, Cambridge, MA, USA) and anti-β-actin (A5441, SIGMA). Proteins were detected using Pierce ECL Western Blotting Substrate (Thermo). Band intensities were compared using the Gel-Pro analyzer software.

### Cell cycle evaluation

Cells were fixed in 70 % ethanol overnight at 4 °C. After PBS washes and incubation in PBS containing 50 μg/mL propidium iodide (PI) and 200 mg/mL RNase A for 30 min in the dark at room temperature, cells were subsequently subjected to flow cytometry analysis. Cell cycle progression was analyzed via fluorescence-activated cell sorting (FACS) on a Partec Flow Max flow cytometer (Partec, Münster, Germany).

### Anoikis evaluation

MHCC-97 L and stably transduced cells were plated in poly-HEMA–coated 6-well plates. After 12, 48 and 72 h of culture, cells were harvested, rinsed with PBS, and apoptosis was evaluated using the Annexin V-PE/7-AAD apoptosis detection kit (KGA1017, KeyGen Biotech, Nanjing, China) on BD FACS Calibur flow cytometer. The percentage of apoptotic cells was derived as the sum of cell fractions displaying early apoptosis (Annexin V-positive) and late apoptosis (7-AAD-positive).

### Transwell migration and invasion assays

A transwell chamber containing an 8-μm pore polycarbonate membrane filter was coated either with (invasion) or without (migration) matrigel (BD, USA), and inserted in a 24-well culture plate. 10^5^ cells/well in 0.2 mL serum-free DMEM was added to the upper chambers of the plates, and DMEM containing 10 % fetal bovine serum in the lower chambers. The plate was then incubated at 37 °C in presence of 5 % CO_2_ for 24 h, and the filter removed. Cells in the upper chamber that did not migrate were scraped away with a cotton swap; trans-membrane cells were fixed in 4 % paraformaldehyde for 30 min and dyed using crystal violet for 25 min. Migrating or invading cells were photographed using an inverted optical microscope (Olympus, Tokyo, Japan). Quantification of migrating or invading cells was determined using the 3-(4,5-dimethylthiazol-2-yl)-2,5-diphenyltetrazolium bromide (MTT) method.

### Scratch test

Cells were resuspended in complete medium, with density adjusted to 1 × 10^6^ cells/ml. Then, 3 ml of cell suspension was added to each well of a 6-well plate. At 80 % confluence, the medium was removed, and a scratch with a standard 200 μl pipette tip was made on the cell layer, equidistant from the dish edges. Wound healing was quantified using the Image J software (National Institutes of Health, Bethesda, MD, USA) as the percentage of wound healing area = [Cell-free area (0 h)- Cell-free area (72 h)]/Cell-free area (0 h).

### Xenograft nude mouse model

Specific-pathogen-free (SPF)-grade adult nude mice (4-6 weeks of age) were housed with pathogen-free fodder, equipment, and environment. Then, 0.2-ml aliquots of non-transduced, and TLN-1 shRNA and scramble shRNA transduced MHCC-97 L cells, respectively (5 × 10^7^cells/200 μl of PBS) were subcutaneously injected at the inguinal region of nude mice in a SPF-grade ultraclean work station. After 20 days, tumor diameters were measured every 3 days with Vernier calipers. Tumor volume (TV) was calculated according to the formula: TV (mm^3^) = d^2^ × D/2, where d and D represent the shortest and the longest diameters, respectively. The mice were sacrificed at 29 days after cell implantation, and the tumors were extracted.

### Statistical analysis

Experimental data were analyzed using the Statistical Package for the Social Sciences (SPSS) 17.0 software (SPSS Inc., Chicago, IL, USA) and Microsoft Office Excel 2010 (Microsoft, Redmond, WA, USA). Data are mean ± standard deviation (SD), and groups were compared using independent samples *t* test and one-way analysis of variance (ANOVA). *P* < 0.05 was considered statistically significant.

## Results

### Establishment of a stable TLN-1 knockdown MHCC-97 L cell line

**Fig. 1:**
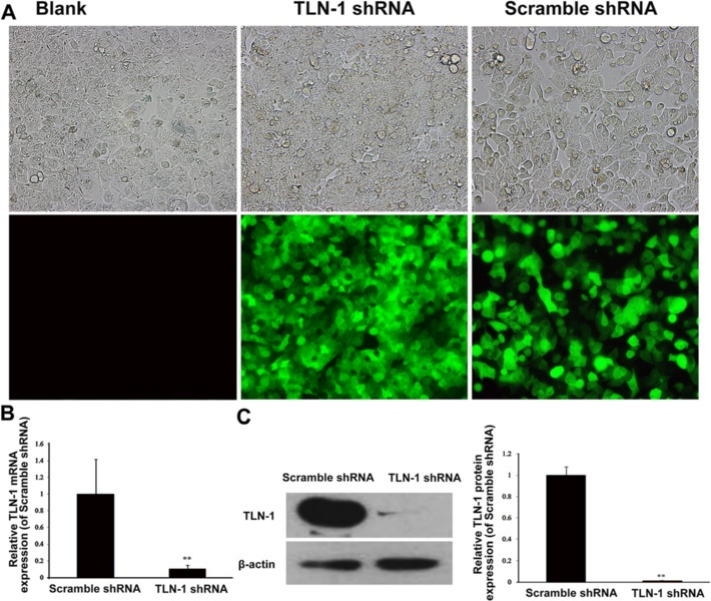
Lentivirus-mediated stable Talin-1 (TLN-1) knockdown in the minimally metastatic HCC MHCC-97 L cells. Blank: non-transduced MHCC-97 L cells; TLN-1 shRNA: MHCC-97 L cells transduced with lentivirus-mediated TLN-1 shRNA; Scramble shRNA: MHCC-97 L cells transduced with lentivirus-mediated scramble shRNA. **a** According to fluorescence microscopy, transduction efficiency in MHCC-97 L cells (the percentage of GFP-positive cells) was >70 % at 72 h (magnification: ×200). **b** TLN-1 mRNA expression levels were determined by real-time RT-PCR at 72 h after transduction, with GAPDH used as an internal control. **c** TLN-1 protein expression levels were determined by Western blot at 72 h after transduction. β-actin was used as an internal control. Data are mean ± Standard deviation (SD). **P < 0.01 *vs*. scramble shRNA

As shown in Fig. 1, a stable TLN-1 knockdown MHCC-97 L cell line was successfully established. According to fluorescence microscopy, transduction efficiency in MHCC-97 L cells (the percentage of GFP-positive cells) was >70 % (Fig. 1a). Furthermore,TLN-1 mRNA and protein levels in the cells transduced with lentivirus-mediated TLN-1-shRNA (experimental group) were markedly reduced compared with the non-transduced cells (blank controls) and cells transduced with lentivirus-mediated scramble-shRNA (negative controls) after 72 h (both *P* < 0.01), as shown by real-time RT-PCR (Fig. 1b) and Western blot (Fig. 1c).

#### Effects of TLN-1 knockdown on TLN-1 and TLN-2 mRNA and protein expressions in MHCC-97 L cells

**Fig. 2:**
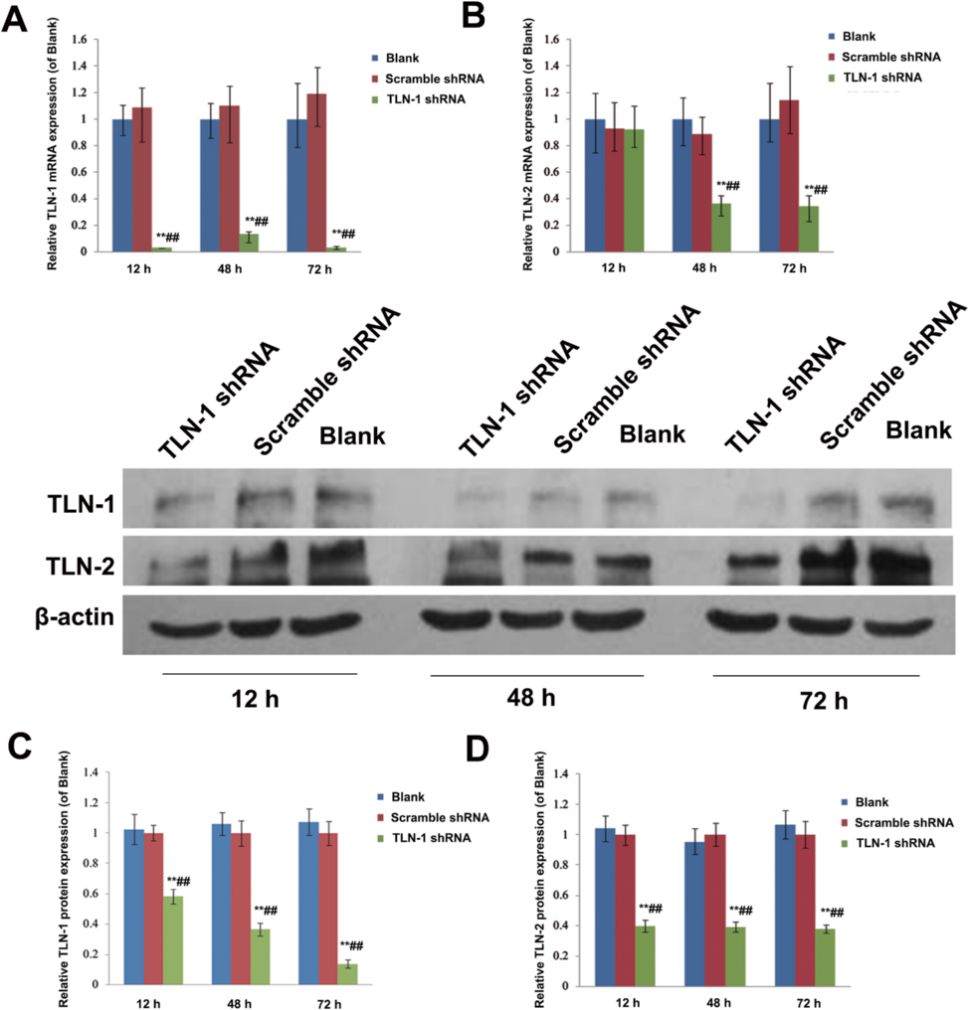
Effects of TLN-1 knockdown on TLN-1 and TLN-2 mRNA and protein expression in MHCC-97 L cells. **a** TLN-1 and (**b**) TLN-2 mRNA expression levels were assessed by real-time RT-PCR at 12 h, 48 h and 72 h after transduction. GAPDH was used as an internal control. **c** TLN-1 and (**d**) TLN-2 protein amounts were determined by Western blot at 12 h, 48 h and 72 h after transduction. β-actin was used as an internal control. Data are mean ± SD. **P < 0.01 *vs*. Blank; ^##^ *P* < 0.01 *vs*. scramble shRNA

The transduced cells were assessed at different time points for their mRNA and protein expression levels. As shown in Fig. 2a, TLN-1 mRNA levels were starkly reduced after gene silencing compared with the blank and negative control groups, at 12, 48, and 72 h (all *P* < 0.01). Although TLN-2 gene expression was less affected than that of TLN-1, significant differences were obtained at 48 and 72 h in the experimental group compared with the blank and negative control groups (both *P* < 0.01, Fig. 2b). The same trend was observed for protein expression, and significantly decreased TLN-1 and TLN-2 were observed at all time points (all *P* < 0.01, Fig 2c and d).

#### Effects of TLN-1/2 knockdown on cell cycle distribution and Anoikis in MHCC-97 L cells

**Fig. 3:**
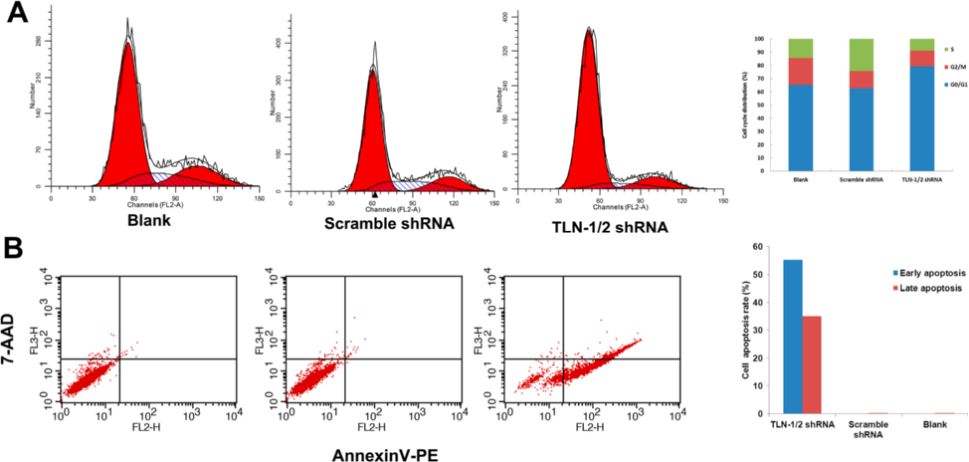
Effects of TLN-1/2 knockdown on cell cycle distribution and Anoikis in MHCC-97 L cells. **a** Cell cycle distribution was determined by flow cytometry using propidium iodide (PI) staining. G0/G1, S and G2/M phase cells were analyzed. **b** Anoikis was determined by flow cytometry using Annexin V-PE/7-AAD staining. Early and late apoptotic cells were analyzed. Data are mean ± SD. **P < 0.01 *vs*. Blank; ^##^ *P* < 0.01 *vs*. scramble shRNA

As shown in Fig. 3a, TLN-1/2 knockdown cells showed significantly more cells in the G0/G1 phase (79.24 %) in comparison with both blank (65.36 %) and negative (62.69 %) control groups; conversely, less cells were found in G2/M and S phases in the experimental group compared with controls. Interestingly, anoikis was enhanced in the experimental group in comparison with controls (*P* < 0.01) (Fig. 3b).

#### Effects of TLN-1/2 knockdown on migration and invasion in MHCC-97 L cells

**Fig. 4:**
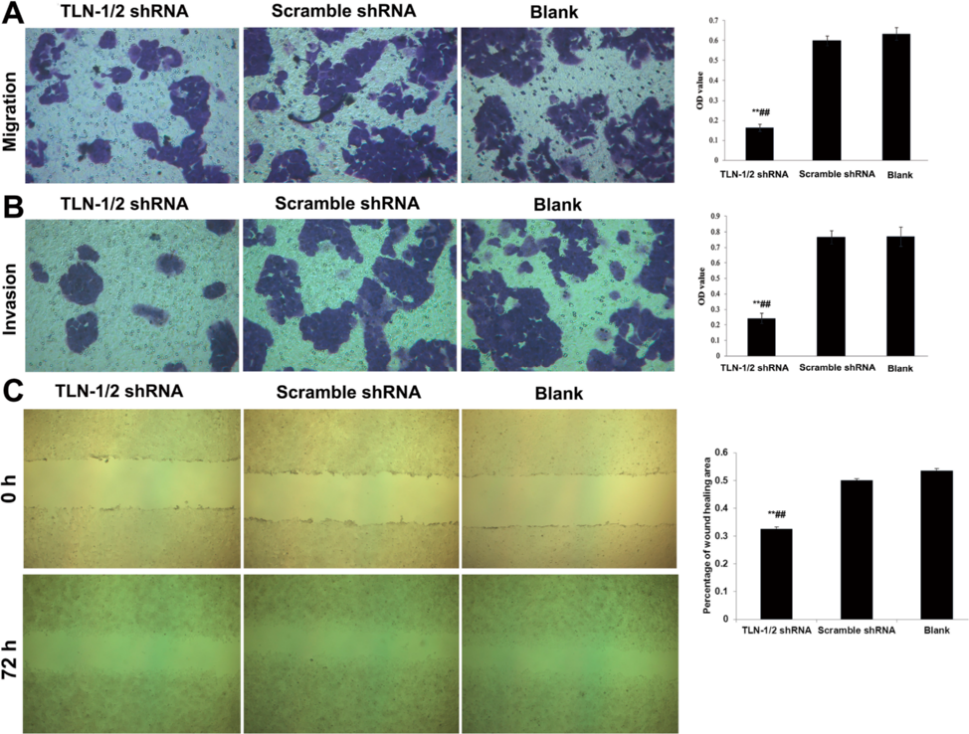
Effects of TLN-1/2 knockdown on migration and invasion in MHCC-97 L cells. **a** Migration and (**b**) invasion abilities of MHCC-97 L cells were examined by transwell assays. The trans-membrane cells were fixed in 4 % paraformaldehyde and dyed using crystal violet (magnification: ×200). Quantification of migrating or invading cells was determined using the MTT assay. OD: Optical density. **c** Migrating ability of MHCC-97 L cells was also determined by wound healing assay at 0 and 72 h after scratching. The tissue monolayer was imaged under light microscopy (magnification: ×200). Data are mean ± SD. **P < 0.01 *vs*. Blank; ^##^ *P* < 0.01 *vs*. scramble shRNA

As shown in Fig. 4a and b, respectively, migration and invasion abilities of MHCC-97 L cells were markedly reduced after TLN-1/2 knockdown (all *P* < 0.01) compared with controls. This was confirmed by the wound healing assay, in which TLN-1/2 knockdown cells showed decreased migration distance compared with both control groups (Percentage of wound healing area: experimental group: 32.6 ± 0.7 % vs. negative controls: 50.1 ± 0.6 % and blank controls: 53.6 ± 0.6 %, both *P* < 0.01) (Fig. 4c).

#### TLN-1/2 knockdown inhibits tumor growth in vivo

**Fig. 5:**
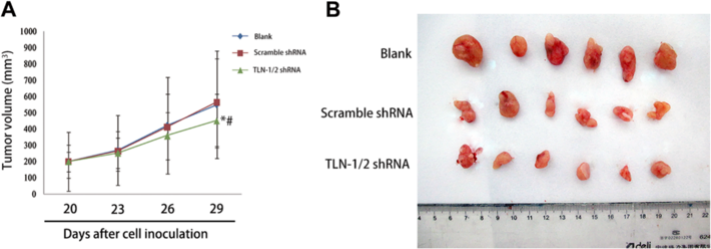
TLN-1/2 knockdown inhibits tumor growth in vivo. MHCC-97 L cells (Blank), MHCC-97 L cells transduced with lentivirus-mediated TLN-1/2 shRNA (TLN-1/2 shRNA) or lentivirus-mediated scramble shRNA (Scramble shRNA) (5 × 10^7^ cells/200 μl of PBS) were subcutaneously injected at the inguinal region of nude mice. **a** Tumor volume (mm^3^) was assessed by calipers every 3 days 20 days after cell inoculation. **b** The mice were sacrificed at 29 days after cell inoculation, and the tumors were extracted. Data are mean ± SD. *P < 0.05 *vs*. Blank; ^#^ *P* < 0.05 *vs*. scramble shRNA

Compared with the blank and negative control groups, TLN-1 shRNA transduced MHCC-97 L cells yielded smaller tumor volume in nude mice after subcutaneous injection, showing weaker cell tumorigenicity. The differences were statistically significant at 29 days after cell inoculation (*P* < 0.05, Fig. 5a). Representative tumors are shown in Fig. 5b.

## Discussion

The roles of TLN-1 and TLN-2 in HCC are not completely understood. We previously assessed five human HCC cell lines and normal liver LO_2_ cells, and showed that TLN-1 protein expression levels in MHCC-97 L cells are highest [8]. In this study we used the lentiviral interference technology to knockdown TLN-1 expression in MHCC-97 L cells, generating a stable transduced cell line. As shown above, TLN-1 knockdown MHCC-97 L cells showed reduced TLN-1 mRNA and protein expressions, as well as arrested cell cycle in the G0/G1 phase, enhanced anoikis, decreased invasion and migration abilities, confirming the involvement of TLN-1 in HCC progression.

Recent studies have demonstrated the complexity of the mammalian TLN-2 gene which may have at least three different protein isoforms expressed in a tissue-specific manner. Additionally, alternatively spliced transcripts have been described for the vertebrate TLN-2 as well as the unique ancestral TLN gene, which is closely related to TLN-2 [6, 17]. Furthermore, TLN-1 and TLN-2 encode proteins with 74 % sequence identity. Thus we further investigated the TLN-2 gene and protein expression by real-time RT-PCR and western blot, respectively, after TLN-1 knockdown. We unexpectedly discovered that TLN-2 expression levels were also decreased in stable TLN-1 knockdown cells. These results indicated that the selected shRNA sequence targeting the TLN-1 gene was directed towards the consensus sequence of TLN-2. Thus, the observed changes of cell cycle distribution, anoikis, migration and invasion, and tumor formation inhibition in vivo were likely associated with the down-regulation of TLN-1 and TLN-2, not just TLN-1.

In this study, TLN-1/2 knockdown resulted in decreased malignancy potential of MHCC-97 L cells in vitro as demonstrated with cell cycle arrest at the G0/G1 phase and enhanced anoikis, as well as decreased migration and invasion capabilities. These corroborate previous reports that TLN-1 is involved in integrin activation [7, 8], which leads to the regulation of adhesion, invasion, proliferation, anoikis, survival, tumor progression and metastasis [7–11, 32]. Interestingly, our in vivo mouse xenograft model confirmed the in vitro findings as smaller tumors were obtained in animals inoculated with the TLN-1/2 knockdown MHCC-97 L cells in comparison with those of the control (blank and negative) groups. Interestingly, KIF14 and TLN-1 inhibition was found to sensitize triple-negative breast cancer (TNBC) cells to therapeutic intervention [19]. However, Talin 1 was recently proposed to be a novel player in the anti-metastatic signaling network of miR-124 [33]. Taken together, these findings suggest that Talin1/2 might regulate HCC invasion and migration through complex mechanisms that are not completely understood.

Although TLN-1 silencing resulted in reduced protein and mRNA expressions of both TLN-1 and 2, it is likely that specific targeting of TLN-2 would have different outcomes. Therefore, we plan in the near future to design specific shRNA aimed at both TLN-1 and TLN-2 to further assess the roles of these two proteins in HCC metastasis and invasion. Alternatively, commercially available TLN-1- and TLN-2-specific monoclonal antibodies will be used to elucidate individual and combined effects of TLN-1 and TLN-2 on primary HCC invasion and migration.

## Conclusion

Our results showed that the levels of both TLN-1 and TLN-2 correlate with tumorigenicity in human HCC, indicating that these molecules constitute useful molecular targets in HCC diagnosis and/or treatment.

## Abbreviations

ANOVA: one-way analysis of variance
DMEM: Dulbecco's Modified Eagle's Medium
ECM: extracellular matrix
FA: Focal adhesion
FACS: fluorescence-activated cell sorting
FAK: focal adhesion kinase
FBS: fetal bovine serum
GAPDH: glyceraldehyde 3-phosphate dehydrogenase
HCC: Hepatocellular carcinoma
KIF14: kinesin family member 14
mRNA: messenger ribonucleic acid
PBS: Phosphate Buffer Saline
PVDF: polyvinylidene difluoride
RT-PCR: real time reverse transcriptase-PCR
SD: standard deviation
SDS-PAGE: sodium dodecyl sulfate-polyacrylamide gel electrophoresis
SPF: specific-pathogen-free
SPSS: Statistical Package for the Social Sciences
TNBC: triple-negative breast cancer
TV: tumor volume
